# Paired whole exome and transcriptome analyses for the Immunogenomic changes during concurrent chemoradiotherapy in esophageal squamous cell carcinoma

**DOI:** 10.1186/s40425-019-0609-x

**Published:** 2019-05-16

**Authors:** Sehhoon Park, Je-Gun Joung, Yang Won Min, Jae-Yong Nam, Daeun Ryu, Dongryul Oh, Woong-Yang Park, Se-Hoon Lee, Yoon La Choi, Jin Seok Ahn, Myung-Ju Ahn, Keunchil Park, Jong-Mu Sun

**Affiliations:** 1 Division of Hematology-Oncology, Department of Medicine, Samsung Medical Center, Sungkyunkwan University School of Medicine, 81 Irwon-ro, Gangnam-Gu, Seoul, 60351 Republic of Korea; 2 Samsung Genome Institute, Samsung Medical Center, Sungkyunkwan University School of Medicine, Seoul, Republic of Korea; 3 Division of Gastroenterology, Department of Medicine, Samsung Medical Center, Sungkyunkwan University School of Medicine, Seoul, Republic of Korea; 4 Department of Radiation Oncology, Samsung Medical Center, Sungkyunkwan University School of Medicine, Seoul, Republic of Korea; 5 Department of Pathology and Translational Genomics, Samsung Medical Center, Sungkyunkwan University School of Medicine, Seoul, Republic of Korea

**Keywords:** Esophageal neoplasms, Chemoradiotherapy, Immune checkpoint inhibitor

## Abstract

**Background:**

The immunogenomic changes triggered by concurrent chemoradiation therapy (CCRT), a standard neoadjuvant treatment for locally advanced esophageal squamous cell carcinoma (ESCC), are unknown. We aimed to analyze the early immunogenomic changes in ESCC induced by CCRT and to correlate them with clinical outcomes.

**Methods:**

We collected biopsy samples from 40 patients with ESCC and the surgical candidates were treated with 5-fluorouracil (5-FU)/Cisplatin and concurrent radiation therapy. Endoscopic biopsy was performed before and after one treatment cycle of 5-FU/Cisplatin and 5 to 18 fractions of radiation. We analyzed immunogenomic changes using paired whole-exome sequencing (*n* = 29) and paired whole-transcriptome sequencing (WTS, *n* = 23). Multiplex immunohistochemistry (IHC) was conducted in four representative pair samples.

**Results:**

Fourteen out of 23 WTS samples (60.8%) showed increased immune scores after CCRT, as calculated by ESTIMATE. The rate of progression-free survival was higher in patients with increased immune scores compared with the remaining patients (83.1% vs. 57.1%, *p* = 0.25). Tumor mutation burden and neoantigen load were significantly reduced after CCRT (*p* < 0.001). We observed no specific correlation with non-synonymous mutations and no changes in the single-nucleotide variant spectrum after CCRT. Post-CCRT samples were enriched in gene sets related to immune signaling pathways, such as interferon gamma signaling and CD28 co-stimulation. Multiplex IHC showed an incremental trend in the proportion of CD4 positive cells in cytokeratin positive region after CCRT. However, CD8, CD20, FOXP1, PD-L1 showed no definitive trend. Proportion of immune cells calculated by CIBERSORT, showed that significant increase in neutrophils after CCRT.

**Conclusions:**

We have comprehensively analyzed the early immunogenomic changes induced in ESCC by CCRT and correlated them with clinical outcomes. Our results provide a potential basis for combining immunotherapy with CCRT for the treatment of ESCC.

**Electronic supplementary material:**

The online version of this article (10.1186/s40425-019-0609-x) contains supplementary material, which is available to authorized users.

## Background

Esophageal cancer, the sixth leading cause of cancer-related deaths, is classified into two main histological subtypes: squamous cell carcinoma (SCC) and adenocarcinoma. Despite the decreased incidence of esophageal squamous cell carcinoma (ESCC) in Western countries, SCC remains prevalent in Asia, Africa, and South America [1]. The combined modality approach of preoperative concurrent chemoradiation therapy (CCRT) and surgery has shown superior clinical outcomes compared with surgery or chemotherapy alone for the treatment of locally advanced ESCC [2–4]. Despite the promising effects of preoperative CCRT, the challenge of cancer relapse after curative resection in patients with ESCC needs to be addressed.

Immune checkpoints are the downregulators of the anti-tumor immune response and their inhibitions are the good treatment strategy as cancer immunotherapy [5, 6]. The synergistic effects of CCRT and immunotherapy have been tested in many clinical trials. In a recent trial, patients with locally advanced non-small cell lung cancer that were treated with durvalumab, an inhibitor of programmed cell death-ligand 1 (PD-L1), after CCRT showed prolonged progression-free survival (PFS) compared with patients that only underwent CCRT [7]. However, the underlying immunogenomic changes induced by CCRT in the tumor and its microenvironment remain unknown. Analysis of such changes may offer additional insights into the synergistic effects of immunotherapy and CCRT and also provide potential predictive or prognostic biomarkers [8].

Here, we conducted an immunogenomic analysis of CCRT-induced changes in ESCC cells using paired whole-exome sequencing (WES) and whole-transcriptome sequencing (WTS). We analyzed previously known immunogenomic markers, such as the proportion of immune cells in the tumor microenvironment [9, 10], somatic mutation profile [11, 12], expression of gene related to cytolytic activity [13, 14], the overall tumor mutation burden (TMB) [15–17], and neoantigens [18, 19], in pre- and post-CCRT samples [20–22]. We also interpreted the clinical implications of these immunogenomic changes based on surgical pathology, immune cell fractions calculated by CIBERSORT [23] and immune scores calculated by ESTIMATE [24]. At the same time, we conducted multiplex immunohistochemistry (IHC) in representative paired samples.

## Methods

### Study flow and patients

**Fig. 1:**
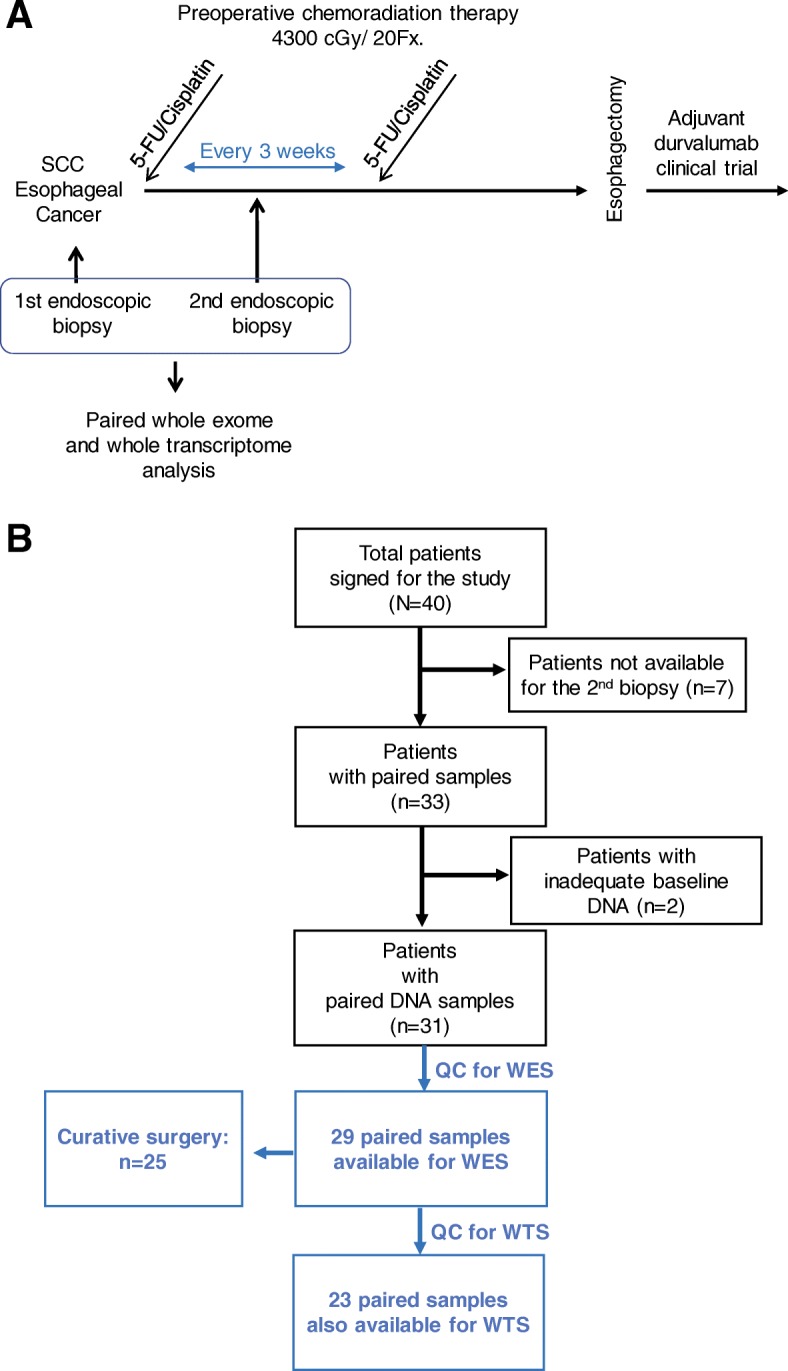
Study flow and CONSORT diagram of the study population. **a** The first endoscopic sample was obtained at the time of diagnosis. The second sample was acquired at 2 to 3 weeks after initiation of concurrent chemoradiation therapy. **b**, Samples were acquired from 40 patients with signed informed consent and prepared for genomic analyses. Twenty-nine patients were available for the paired whole-exome sequencing (WES), and 23 patients were available for the paired whole-transcriptome sequencing (WTS). A total of 25 patients underwent curative surgery. Abbreviations: SCC, squamous cell carcinoma; QC, quality control

Patients with esophageal cancer at clinical stages T1b-T4a and N0 or N+ were evaluated for surgery by a multidisciplinary team that included a medical oncologist, a radiation oncologist, a radiologist, and a thoracic surgeon. The surgical candidates (*n* = 40) underwent neoadjuvant chemoradiotherapy. Radiation therapy was delivered with a dose of 4300–4400 cGy in daily 210–215 cGy per fraction over 4 to 5 weeks. Concurrent chemotherapy of 5-FU (4000 mg/m^2^ over 4 days) and cisplatin (60 mg/m^2^ on day 1) were administered every 3 weeks for up to two cycles during RT. Endoscopic biopsies were performed at the time of initial diagnosis and 2 to 3 weeks after the start of preoperative CCRT (Fig. 1a). This genomic analysis study was approved by the institutional review board of the Samsung Medical Center (IRB no. SMC-2013-10-112) and written informed consent was obtained from all enrolled patients.

### Isolation of genomic DNA and RNA

Genomic DNA (gDNA) and RNA were purified from cancer tissues using the AllPrep DNA/RNA Mini Kit (Qiagen, USA). gDNA from peripheral blood was extracted using the QIAamp DNA Blood Mini Kit (Qiagen). gDNA concentration and purity were measured using a NanoDrop 8000 UV-Vis Spectrometer (Thermo Scientific Inc., USA) and a Qubit 2.0 Fluorometer (Life Technologies Inc., USA). To measure DNA degradation, median DNA size and ΔCt values were measured using a 2200 TapeStation Instrument (Agilent Technologies, USA) and real-time polymerase chain reaction (PCR; Agilent Technologies), respectively. RNA concentration and purity were measured using the NanoDrop and Bioanalyzer (Agilent Technologies).

### Whole-exome sequencing

High quality gDNA in each sample was sheared with an S220 ultra-sonicator (Covaris, USA) and used to construct a library with the SureSelect XT Human All Exon v5 and SureSelect XT reagent kit, HSQ (Agilent Technologies), according to the manufacturer’s protocol. This kit is designed to enrich 335,756 exons from 21,058 genes, covering ~ 71 Mb of the human genome. Enriched exome libraries were multiplexed and sequenced on the HiSeq 2500 platform (Illumina, USA). A paired-end DNA sequencing library was prepared via gDNA shearing, end-repair, A-tailing, paired-end adaptor ligation, and amplification. After hybridizing the library with bait sequences for 16 h, the captured library was purified and amplified with an indexing barcode tag, and library quality and quantity were assessed using a 2200 TapeStation Instrument and Qubit 2.0 Fluorometer, respectively. The exome library was sequenced using the 10-bp paired-end mode of the TruSeq Rapid PE Cluster kit and the TruSeq Rapid SBS kit (Illumina).

### Exome sequence data analysis

The University of California Santa Cruz hg19 reference genome (downloaded from http://genome.ucsc.edu) was used to align sequencing reads via the Burrows-Wheeler Aligner v. 0.6.2(15) with default settings. PCR duplications were marked using Picard-tools-1.8 (http://broadinstitute.github.io/picard/) and data cleanup was achieved using GATK-2.2.9 [25]. Point mutations were identified with the MuTect tool (https://github.com/broadinstitute/mutect) in paired samples. Indels were detected by VarScan2 (http://varscan.sourceforge.net/). Annovar was used to annotate variants. Signature analysis of mutational processes was carried out using the deconstructSigs tool [26]. Copy number variations were identified by EXCAVATOR [27]. Significant focal somatic copy number alterations were summarized by GISTIC analysis [28]. Tumor purity was estimated by PyLOH [29].

### RNA sequencing

Library construction for RNA sequencing (RNA-seq) was performed using a Truseq RNA Sample Preparation v2 Kit (Illumina). Isolated total RNA was used in a reverse transcription reaction with poly (dT) primers, using SuperScriptTM II Reverse Transcriptase (Invitrogen/Life Technologies, USA), according to the manufacturer’s protocol. An RNA-seq library was prepared via cDNA amplification, end-repair, 3′ end adenylation, adapter ligation, and amplification. Library quality and quantity were measured using the Bioanalyzer and Qubit. Sequencing of the RNA library was carried out using the 100-bp paired-end mode of the TruSeq Rapid PE Cluster Kit and the TruSeq Rapid SBS Kit (Illumina).

### RNA-Seq data analysis

The reads from the FASTQ files were mapped against the hg19 human reference genome using TopHat version 2.0.6 (http://ccb.jhu.edu/software/tophat/index.shtml). Raw read counts mapped to genes were measured using the BAM format file by HTSeq version 0.6. 1 [30]. A total of 18,161 coding genes were analyzed for transcript abundance and poorly expressed genes were eliminated based on the criteria of a maximum read count > 20 for all samples. Read counts were normalized using the Trimmed Mean of M-values normalization method. Differentially expressed genes were identified using the DESeq R package (www.huber.embl.de/users/anders/DESeq/). A gene set enrichment analysis (GSEA) [31] was conducted to analyze up- or down-regulated gene sets in specific groups of ESCC samples. Stromal and immune scores based on WTS were calculated using ESTIMATE [24]. Fractions of immune-associated cell types were calculated by CIBERSORT using RNA-seq expression profiles [23]. The immune cytolytic activity (CYT) was measured by the geometric mean of *GZMA* and *PRF1* expression values in TPM [13].

### Cancer cell fraction measurement

Cancer cell fractions (CCF) were measured by PyClone, which de-convolves tumor sequences into sub-clones based on a hierarchical Bayesian clustering model [32]. Input data were generated from somatic single-nucleotide variants (SNVs) detected by MuTect and corresponding copy number variations. SNVs were grouped into clusters with similar CCF values.

### Tumor mutation burden and prediction of candidate neoantigens

TMB was measured by the number of somatic single nucleotide variants and indel mutations per megabase in the coding region [33]. Somatic single nucleotide variants include nonsynonymous as well as synonymous mutations. Non-coding alterations were not counted. In addition, known somatic alterations in COSMIC and truncations in tumor suppressor genes were excluded from the count.

Neoantigens were predicted using MuPeXI v.1.1.3 [34]. The three types of human leukocyte alleles (HLA-A, −B, and -C) were identified from the WTS data of each patient using seq2HLA [35]. Somatic mutations, gene expression counts, HLA types for each patient, and peptide lengths (8–11 mer) were provided as input for MuPeXI. Peptides with a half maximal inhibitory concentration (IC50) value ≤500 nM were considered to have a high binding affinity for the major histocompatibility complex (MHC). Expressed mutant peptide sequences with an IC50 value of ≤500 nM that showed binding affinity above normal were picked as candidate neoantigens.

### Multiplex immunohistochemistry (IHC) and analysis

4-μm sections of specimens were cut from formalin-fixed paraffin-embedded (FFPE) blocks. Slides were heated for at least one hour in a dry oven at 60 °C and dewaxed using xylene, then followed by multiplex immunofluorescence staining with a Leica Bond Rx™ Automated Stainer (Leica Biosystems, Newcastle, UK). Briefly, the slides were baked for 30 min and dewaxed with Leica Bond Dewax solution (#AR9222, Leica Biosystems), followed by antigen retrieval with Bond Epitope Retrieval 2 (#AR9640, Leica Biosystems) in a pH 9.0 solution for 30 min. The first primary antibodies for CD4 (ab133616, Abcam, dilution 1:100) were incubated for 30 min, followed by detection using the Polymer HRP Ms. + Rb (ARH1001EA, Perkin-Elmer) for 10 min. Visualization of CD4 was accomplished using Opal 570 TSA Plus (dilution 1:150) for 10 min, after which the slide was treated Bond Epitope Retrieval 1 (#AR9961, Leica Biosystems) for 20 min to remove bound antibodies before the next step in the sequence. In a serial fashion, CD20 (ab9475, Abcam, ARH1001EA, Perkin-Elmer, Opal 520 TSA Plus), FOXP3 (ab20034, Abcam, ARH1001EA, Perkin-Elmer, Opal 690 TSA Plus), PD-L1 (13684S, Cell Signaling, ARH1001EA, Perkin-Elmer, Opal 620 TSA Plus), CD8 (MCA1817T, BIO-RAD, ARH1001EA, Perkin-Elmer, Opal 480 TSA Plus) and CK (M3515, Dako, ARH1001EA, Perkin-Elmer, Opal 780 TSA Plus) was stained. Nuclei were subsequently visualized with DAPI, and the section was coverslipped using HIGHDEF® IHC fluoromount (ADI-950-260-0025, Enzo).

Slides were scanned using the PerkinElmer Vectra 3.0 Automated Quantitative Pathology Imaging System (Perkin-Elmer, Waltham, MA), and images were analyzed using the inform 2.2 software and TIBCO Spotfire™ (Perkin-Elmer, Waltham, MA). Each cell was identified by detecting nuclear spectral elements (DAPI). All the immune cell populations from each panel were characterized and quantified using the cell segmentation tool by the InForm image analysis software. All cells in each slide were designated as positive or negative for each antibody, and the data were categorized and exported to an xls file for analysis. We used the Spotfire™ program after tissue and cell segmentation and expression intensity was compared and then judged based on the cut-off value. The numbers of CD4, CD20, FOXP3, PD-L1, CD8, and CK positive cells were counted in each slide.

### Statistics

The two-sided t-test was used for comparisons of tumor purity, mutation burden, immune and stromal score, cytolytic score, fraction of immune cell from the pre- and post-CCRT samples. The Kaplan-Meier curves and log-rank test was used for the survival analysis. *P*-value less than 0.05 was considered as a statistically significant.

## Results

### Baseline demographics

From a total of 40 study participants, 29 and 23 patients participated in paired DNA analysis and whole-transcriptome analysis, respectively (Fig. 1b). The pre-treatment clinical stages were IVA (*n* = 4), III (*n* = 21), and II (*n* = 4). Of the 29 patients that enrolled for paired genomic analysis, four patients did not undergo surgery due to disease progression (*n* = 2), refusal of surgery, and failure to follow-up. Surgical samples after CCRT (*n* = 25) showed post-neoadjuvant stages of IVA (*n* = 2), IIIB (*n* = 3), IIIA (*n* = 7), II (*n* = 1), I (*n* = 7) and 5 cases of pathologic complete response (pCR). The first biopsy was conducted at a median time of 4 days (range 1–14) before CCRT and the second biopsy was conducted at a median of 18 days (range 4–24) after CCRT. The resection margins were negative for all samples. Seventeen patients are currently enrolled in other clinical trial, which aims to study the effects of randomized adjuvant durvalumab treatment versus placebo (NCT02520453; Additional file 1: Table S1) [36].

### Changes in the somatic mutation landscape and copy number alteration in pre- and post-CCRT samples

**Fig. 2:**
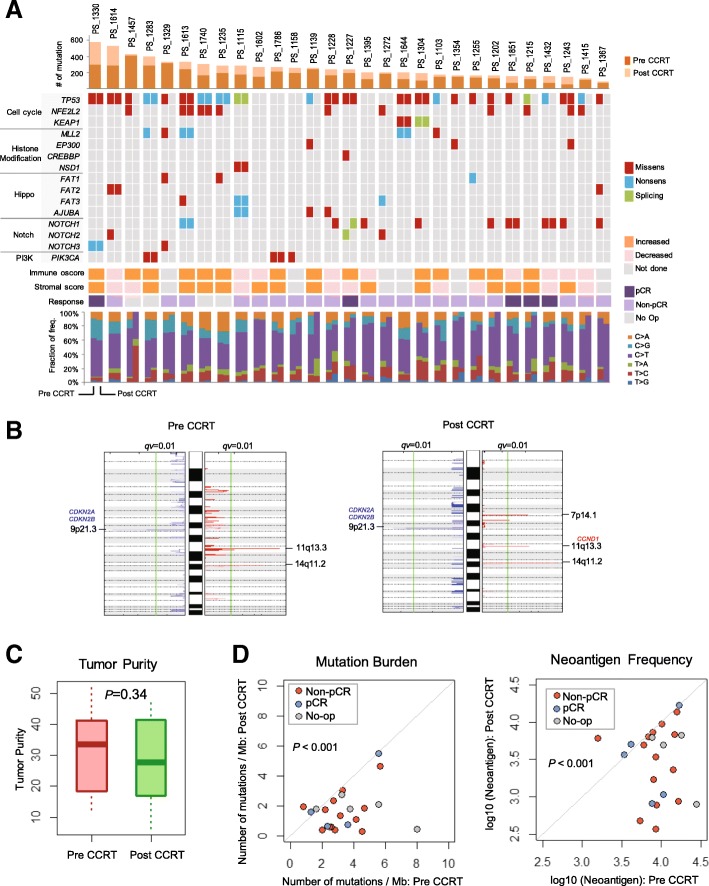
Tumor mutation profile and changes in copy number, mutation burden in pre- CCRT and post-CCRT biopsy samples. **a**, Heatmap of somatic mutations (including missense, nonsense, and splicing) detected in 29 study patients. Each patient had a paired column (1st: Pre-CCRT and 2nd: Post-CCRT). Samples were annotated for mutation quantity, immune or stromal score changes, and surgical pathology. Each bar portrayed in the bottom represents the proportion of each type of substitution. **b**, Copy number alteration was analyzed from pre- and post-samples. **c**, Tumor purity was calculated by whole-exome sequencing. **d**, Comparison of mutation burden and neoantigen frequency between pre-CCRT and post-CCRT samples (*n* = 29). Abbreviations: CCRT, concurrent chemoradiation therapy; pCR, pathologic complete response

We compared the somatic mutation landscape between pre- and post-CCRT samples, and analyzed representative genes related to cell cycle, histone modification, Hippo pathway, Notch pathway, and the PIK3CA pathway (Fig. 2a). Twenty-four pre-CCRT samples had missense, nonsense, or splicing mutations in the tumor suppressor gene, *TP53*, and these alterations were maintained in 11 samples after CCRT. Although nine pre-CCRT samples had a missense mutation in the nuclear factor erythroid 2 like 2 gene, *NFE2L2*, only two post-CCRT samples retained that mutation. SNV analysis showed that transition mutations, specifically C to T or G to A, were prominent in all samples. Comparison of copy number amplification and deletion regions between genomes of pre-CCRT and post-CCRT samples (Fig. 2b), showed that copy number at chromosome 7p14.1 was amplified only in post-CCRT samples. Additionally, the region harboring the cyclin D1 (*CCND1*) oncogene was amplified and the region harboring the tumor suppressor genes, Cyclin Dependent Kinase Inhibitor 2A (*CDKN2A)* and *CDKN2B*, was deleted in both pre- and post-CCRT samples.

### Analysis of TMB, immune and stromal score profile, and immune cell composition in the tumor microenvironment in pre- and post-CCRT samples

**Fig. 3:**
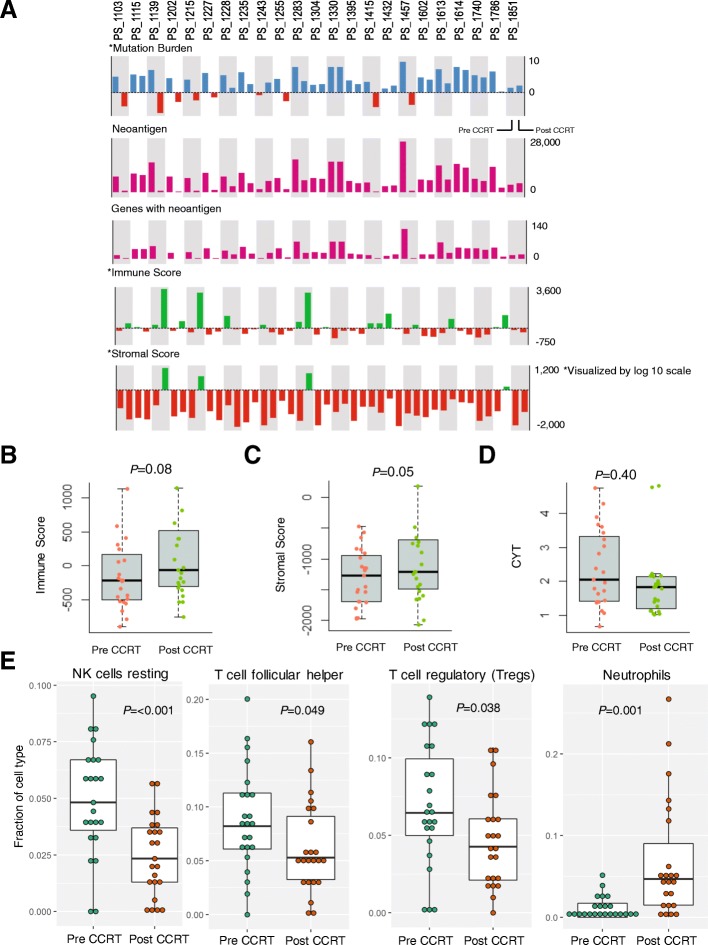
Comparison of changes in immune cell fractions, and immune, stromal, and immune cytolytic activity scores between pre-CCRT and post-CCRT samples. **a** Details regarding the change in each sample based on pre-CCRT and post-CCRT samples. Comparison of the immune and stromal score (**b**) and (**c**), and immune cytolytic (CYT) activity (**d**), in samples available for paired whole-transcriptome sequencing (*n* = 23).**e,** The fraction of immune cells showing statistically significant changes (*n* = 23). Abbreviations: CCRT, concurrent chemoradiation therapy, CYT, cytolytic activity

We found no difference in tumor purity, between pre- and post-CCRT samples (Fig. 2c). This allowed us to analyze the TMB and neoantigen load using WES and WTS data. We found that the TMB and neoantigen load were significantly lower in post-CCRT samples (*p* < 0.001) compared with pre-CCRT samples (Fig. 2d). Using ESTIMATE (Estimation of STromal and Immune cells in MAlignant Tumor tissues using Expression data), we found that 14 post-CCRT samples (60.9%) showed an increased immune score compared with pre-CCRT samples. Of those 14 post-CCRT samples, 10 also showed a concurrent increase in the stromal score (Fig. 3a). Therefore, CCRT leads to increased immune and stromal scores in the tumor tissue (Figs. 3b and c). No difference in immune CYT was observed between pre- and post-CCRT samples (Fig. 3d). Using CIBERSORT, we analyzed the changes in the population of immune cell types before and after CCRT. We found that the numbers of resting natural killer (NK)/T-cells (*p* < 0.001), follicular helper T-cells (*p* = 0.049), and regulatory T-cells (*p* = 0.038) significantly decreased after CCRT. However, the population of neutrophils was significantly increased in the post-CCRT samples (*p* = 0.001; Fig. 3e). We found no difference in the abundance of major immune cells such as B-cells, CD4 T-cells, CD8 T-cells, and M0, M1, and M2 macrophages between pre- and post-CCRT cells.

### Multiplex immunohistochemistry results in pre- and post- CCRT samples

**Fig. 4:**
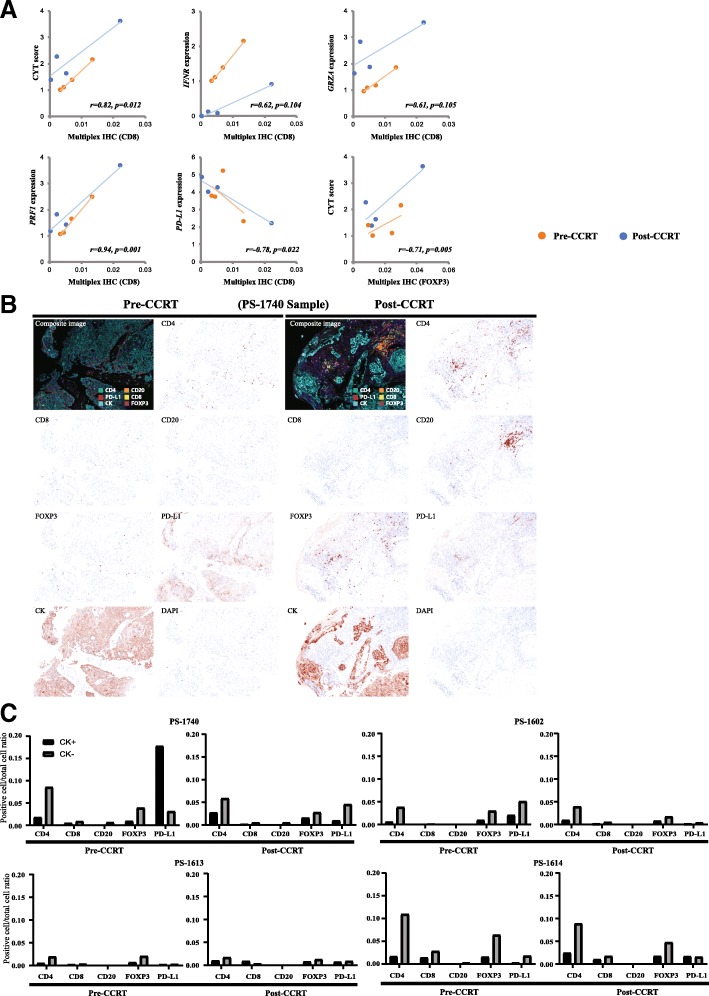
**a** Correlation between multiplex IHC result and expression of genes and gene sets. CYT score, PERF1, PD-L1 were correlated with CD8 positive cells and CYT was correlated with FOXP3 positive cell by multiplex IHC. Pearson’s r and *P*-value is calculated using both pre- and post- CCRT sample. **b** Represent images from multiplex IHC (PS-1740) showing result from pre-CCRT and post-CCRT samples. **c** The proportion of cells per total cell in shown based on cytokeratin positivity

We found a strong correlation between outcomes from gene expression profile and multiplex IHC. Multiplex IHC for CD8 was significantly correlated with CYT score (*p* = 0.012). Especially *PRF1* (Perforin 1) expression among CYT genes was significant (*p* = 0.001) while *GRZA* (Granzyme A) was not significant (*p* = 0.105). IFN-r was also correlated with CD8 (*p* = 0.104), and PD-L1 expression was negatively correlated with CD8 (*p* = 0.022) (Fig. 4a). Multiplex IHC is conducted in four patients available for both pre- and post- CCRT samples (Fig. 4b, Additional file 2: Table S2). Interestingly, all the samples showed an incremental trend in CD4 cell proportion after the CCRT. The proportion of cell expressing CD8, CD20, FOXP3, PD-L1 showed no definitive trend. (Fig. 4c). TMB was also highly correlated with CD8, but it was not significant (*r* = 0.69, *p* = 0.059) (data not shown).

### Tissue characteristics and survival analysis of patients with an increased immune score after CCRT

**Fig. 5:**
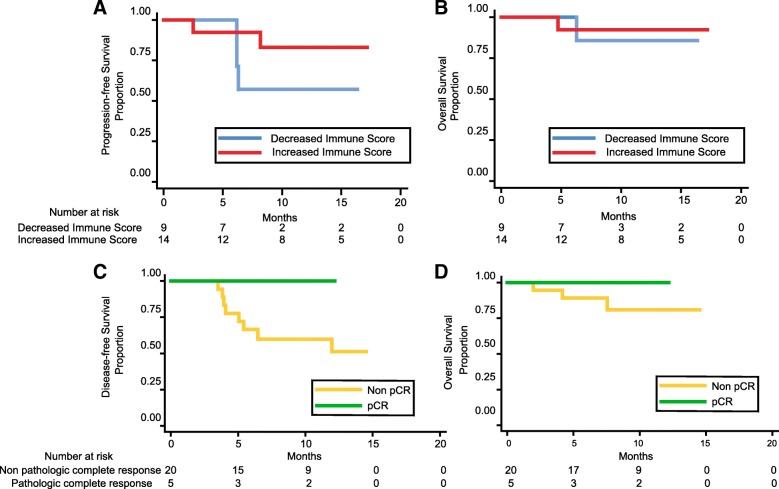
Survival analysis based on immune score changes and pathologic complete response. Twenty-three patients with paired immune score showed (**a**)**,** progression-free survival: hazard ratio (HR) 0.362, 95% confidential interval (CI) (0.060–2.184), *p* = 0.248; and (**b**)**,** overall survival: HR 0.585, 95% CI (0.036–9.381), *p* = 0.702. **c,** Disease-free survival: HR 4.69e-16, 95% CI (0-NR), *p* = 0.210 (**d**)**,** and overall survival: HR 4.84e-16, 95% CI (0-NR), *p* = 0.465 was shown in 25 patients who had curative surgery. HR and *P* calculated by log-rank test. Abbreviations: NR, not reached; pCR, pathologic complete response

The 14 post-CCRT samples that showed increased immune scores also showed an increased abundance of neutrophils and a decrease in the number of follicular helper T-cells, regulatory T-cells, and resting NK/T-cells compared with the baseline pre-CCRT samples. We found that pre-CCRT samples with a higher proportion of M0 macrophages and lower resting mast cells were likely to show an increase in immune score after CCRT (Additional file 3: Table S3). Survival analysis showed extended PFS and overall survival (OS) in patient subgroups with increased immune scores (12-month PFS rate: 83.1% vs. 57.1%, *p* = 0.248; 12-month OS rate: 92.3% vs. 85.7%, *p* = 0.702) compared with the remaining subgroups (Fig. 5a and b). To validate the strength of these potential prognostic factors, we analyzed patient survival using pCR, a well-known prognostic factor for ESCC [37]. Disease free survival (DFS) and OS were extended in patients that showed pCR after CCRT (12-month DFS rate: 100.0% vs. 62.2%, *p* = 0.210; 12-month OS rate: 100.0% vs. 82.2%, *p* = 0.465), which was similar to the difference in survival rates analyzed according to the change of immune score (Figs. 5c and d).

### Differentially expressed gene analysis and gene set enrichment analysis

**Fig. 6:**
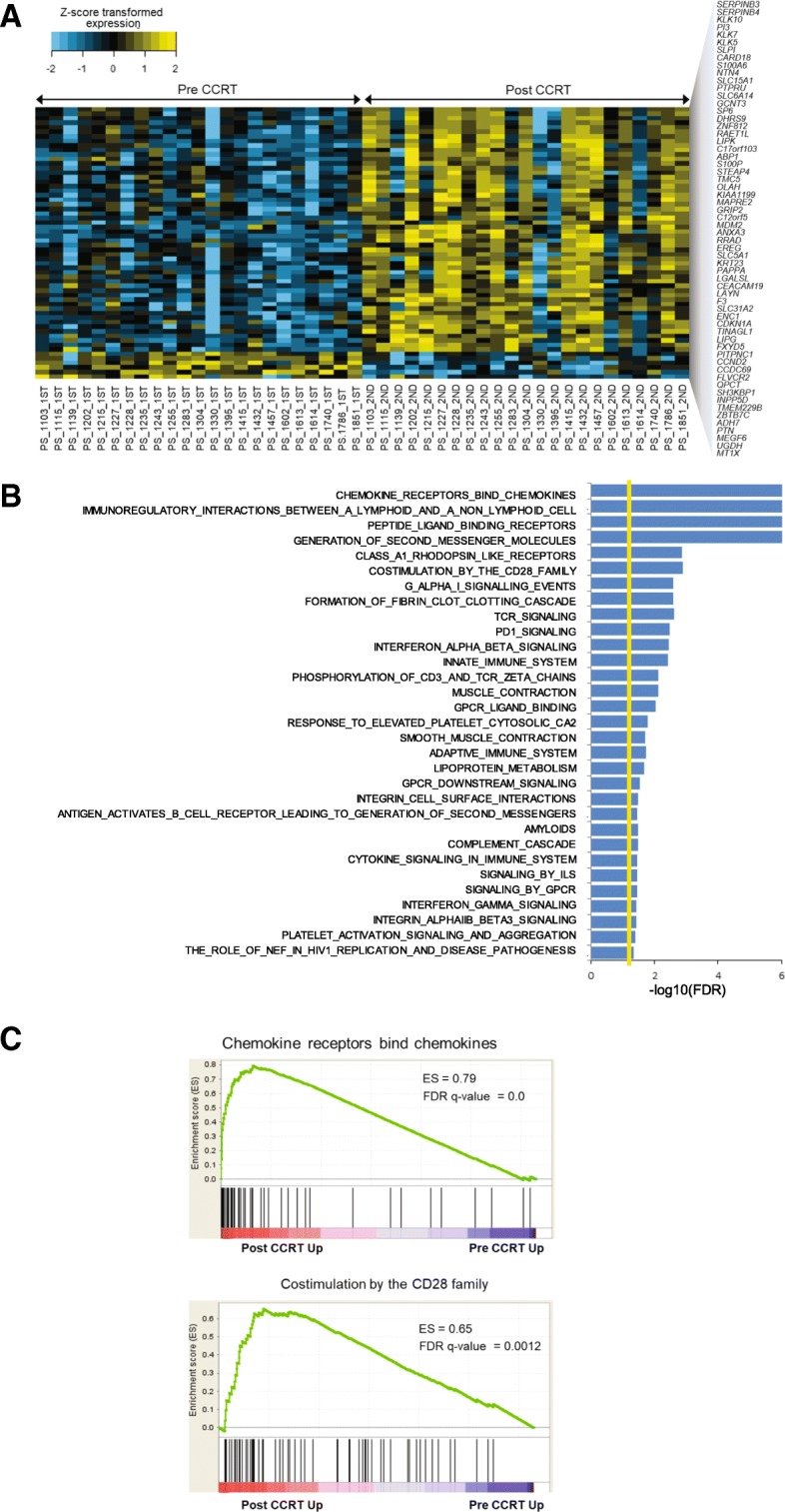
Differentially expressed genes and gene set enrichment analysis in pre- and post-CCRT samples**. a**, Heatmap comparison of 60 significant genes (> 2-fold change & adjusted *p* < 0.01) between pre- and post-CCRT samples. **b**, Enriched differentially expressed gene sets between pre-CCRT and post-CCRT. **c,** Representative gene set enrichment analysis of differentially expressed genes in pre- and post-CCRT samples

Sixty genes that satisfied pre-defined criteria (> 2-fold change and adjusted *p* < 0.01) were identified by differentially expressed gene analysis in pre- and post-CCRT tissue samples (Fig. 6a and Additional file 4: Table S4). We found that the expression of cell cycle-related tumor suppressor genes, *CCND2* and *CDKN1A*, was increased after CCRT. Gene set enrichment analysis showed that several immune-related gene sets, such as those involved in interferon gamma signaling, cytokine signaling, adaptive immune system, innate immune system, PD1 signaling, T-cell receptor signaling, and CD28 co-stimulation were enriched in post-CCRT samples (Figs. 6b and c).

### Correlation of genomic profile with treatment outcome

**Fig. 7:**
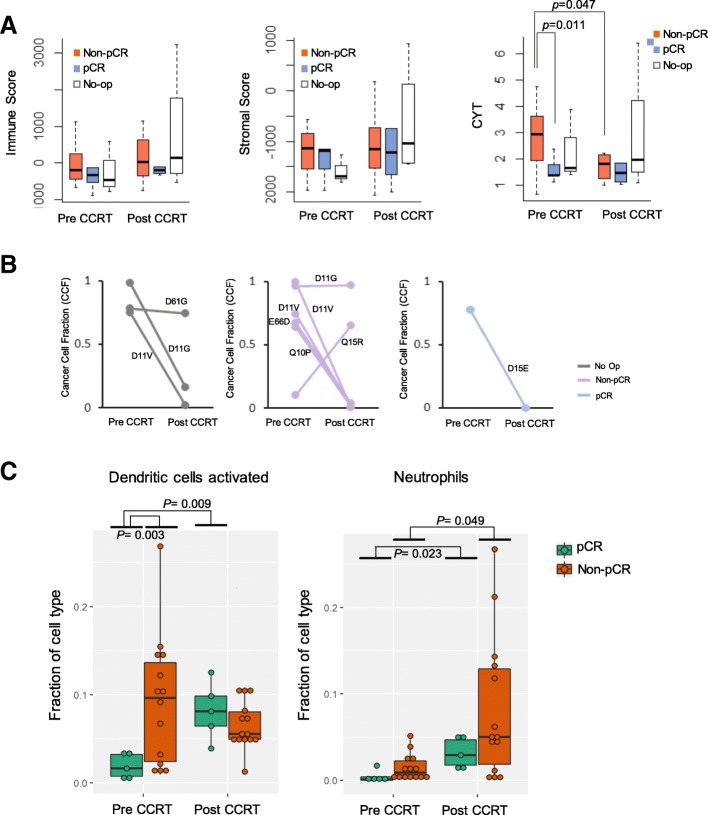
Comparison of changes in the immune, stromal, and immune cytolytic activity scores and immune cell fractions between pre- CCRT and post-CCRT samples based on their surgical pathologic profile. **a**, Immune and stromal scores, and immune cytolytic (CYT) activity in pathologic complete response (pCR), non-pCR, and no-operations (Op) samples. **b**, Somatic mutation changes in *NFE2L2* using cancer cell fraction measurements based on the surgical pathologic profile.**c,** Changes in immune cell fractions based on the surgical pathologic profile

To analyze the genomic profile of the ESCC tissue samples based on their surgical pathology, we compared pCR (*n* = 5) and non-pCR (*n* = 20) subgroups using pre- andpost-CCRT samples. We observed no significant difference in immune and stromal scores between the two subgroups in pre-CCRT samples. However, the pCR subgroup showed significantly lower immune CYT compared with the non-pCR subgroup (*p* = 0.011) in pre-CCRT samples. We found a prominent decrease in CYT in the non-pCR group (*p* = 0.047) after CCRT, but no change in CYT in the pCR group compared with pre-CCRT samples (Fig. 7a). We also did not find any predictive somatic mutation markers in the pCR subgroup. *NFE2L2*, a gene frequently found to be mutated in our pre-CCRT samples, mostly disappeared after CCRT (Fig. 3a). CCF estimation analysis showed that *NFE2L2* p.D15E was the only unique variant in pCR samples. However, the proportion of cancer cells with specific mutation failed to specifically correlate with the pathologic response (Fig. 7b). Analysis of changes in the population of immune cell types showed a significant increase in activated dendritic cells after CCRT in the pCR subgroup compared with pre-CCRT samples. Neutrophils were significantly increased in number in both pCR and non-pCR subgroups after CCRT compared with pre-CCRT samples (Fig. 7c and Additional file 5: Table S5). However, analysis of individual gene expression patterns revealed no specific pattern in the pCR and non-pCR subgroups before and after CCRT (Additional file 6: Table S6 and Additional file 7: Fig. S1).

In addition, we looked into the difference in immune profiles between samples from inoperable patients (*n* = 2) and the patients who received surgery (*n* = 25). In pre-CCRT samples from inoperable patients showed significantly lower stromal and CYT scores compared with patients who received surgery (*P* = 0.0014 and *P* = 0.012, respectively). A similar pattern in immune scores was shown despite not significant (Additional file 8: Fig. S2). Looking into the immune cell compositions between two groups, a fraction of resting NK cells and regulatory T cells in pre-CCRT samples were significantly higher in inoperable patients compared to the patients who conducted surgery (*P* = 0.037 and *P* = 0.005, respectively). There was no significant difference in TMB and neoantigen load between both groups.

## Discussion

Recent advances in modern sequencing and analytical tool-kits have resulted in the evolution of immunogenomics as a critical component of cancer immunotherapy. Immunogenomic studies help to understand the mechanisms that control therapy response and resistance to immune response shaped by the tumor and its microenvironment. In this study, we have comprehensively analyzed the changes in the immunogenomic profile of ESCC in response to CCRT.

Using our sequencing dataset, we analyzed the genomic profiles of patients with ESCC to identify subgroups that could benefit from CCRT. We found that patient subgroups with increased immune scores after CCRT showed favorable survival outcomes. However, the elevated immune score could be primarily due to an increase in the abundance of neutrophils, not adaptive immune cells, in the tumor microenvironment. Therefore, our results suggested that neutrophils were the primary modulators of immungenomic changes during CCRT which also consequences comparably favorable survival outcomes.

Previous studies [38–40] have suggested that the molecular signature of *NFE2L2* (*NRF2*), a master transcriptional regulator of stress response, serves as a predictive marker for esophageal tumor response to CCRT. A gain-of-function *NRF2* mutation confers resistance to therapy in ESCC cells [39]. However, our results showed no significant correlation between *NFE2L2* missense mutations and the pCR of the study population (pCR rate of 14.3% in patients with *NFE2L2* mutations vs. 20.0% in the remaining patients). It is possible that the missense mutations observed in our study population failed to affect *NFE2L2* function and further analysis is needed to study the functional alteration of the mutated forms of *NFE2L2* in the ESCC tissue samples.

The tumor microenvironment contains diverse immune cell types in addition to tumors cells and its nature and composition change over time with treatment [41, 42]. From our previous study, it is known that PD-L1 expression elevated in ESCC samples that received preoperative CCRT compared to the CCRT naïve sample [43]. These findings reflect that CCRT induces immune checkpoint protein expression in tumor which we could also expect alteration in immune cell composition in tumor microenvironment lead by upregulation of PD-L1. We found an enrichment of neutrophils in the tumor microenvironment after CCRT (Fig. 3e). However, we failed to see an increase in the number of adaptive immune cells needed for anti-tumor immune response, such as activated CD8 T-cells and dendritic cells, after CCRT. CYT, which is indicative of activated CD8 T-cells, was also not elevated after CCRT (Fig. 3d). Radiation is known to induce an inflammation response by damaging tumor endothelial cells and triggering inflammatory cytokine signaling (via Interleukin 1 and the tumor necrosis factor) [44, 45]. Radiation also induces the recruitment of circulating immune cells and increases antigen exposure to initiate an adaptive immune response [46]. Our results on neutrophil enrichment after CCRT suggested that chemoradiation, unlike radiation only, induce non-specific inflammation rather than an adaptive immune response in the tumor microenvironment of ESCC.

High TMB and neoantigen load in a tumor can generate T-cell responses that recognize and eradicate tumor cells [47]. Clinical trials have shown that high TMB increases the efficacy of immune checkpoint blockades in cancer immunotherapy [16]. We found that TMB and neoantigen load were significantly reduced (*p* < 0.001) in ESCC samples after CCRT. Radiotherapy is known to induce antigen presentation [46]. However, our results showed that underlying tumor mutation burden, which hypothetically shows positive correlation to tumor antigen presentation, were lowered after chemoradiation.

Based on our results that CCRT decreases the TMB and induces non-specific inflammation in ESCC cells, it is possible that the immune cell priming and reinvigoration induced by immune checkpoint inhibitors can have a greater impact when immunotherapy is combined before or at the time of initiating CCRT. Patients with non-small cell lung cancer (NSCLC) that undergo consolidation therapy with anti-PD-L1 inhibitor (durvalumab) following completion of CCRT show increased survival [7]. Concurrent durvalumab treatment with CCRT, starting with CCRT not after CCRT, is currently being tested for improved synergistic effect and survival benefit compared to previous result in same NSCLC population (NCT03519971) [48]. Also, current clinical trials are testing combination therapies in which immunotherapy is initiated one or two weeks prior to CCRT [49–51]. Results from these clinical trials may validate our hypothesis.

One of the challenges that we faced during this study was the selection of an optimal timepoint for the second biopsy to best identify the immunogenomic changes caused by CCRT. We had initially designed the study to compare tissue samples from patients with ESCC at the time of diagnosis and post-surgical samples from patients that had undergone CCRT. However, we were unable to perform genomic analyses on samples that were exposed to the entire CCRT regimen due to extensive tumor necrosis. Therefore, we conducted the second biopsy within 2 to 3 weeks after the initiation of CCRT. Due to the reason, small number of samples were available for the multiplex IHC which weakens the representative value of its result and showed inconsistent result in some result such as trend of CD4-cells before and after the CCRT. Future studies should confirm whether our timepoints were optimal for the evaluation of immunogenomic changes in the tumor and its microenvironment.

In conclusion, our study is the first to demonstrate the underlying genomic changes caused by CCRT. It will provide a basis for further genomic studies in patients that undergo CCRT and guide clinical trials that test combination therapy treatments of immunotherapy and CCRT.

## Additional files

Additional file 1: **Table S1.** Clinical information of study population (XLS 2133 kb)

Additional file 2: **Table S2.** Samples available for the Multiplex IHC (XLSX 11 kb)

Additional file 3: **Table S3.** The difference between groups for relative fractions of major immune cell types compared by increase in immune score calculated by ESTIMATE. The significance was measured by a t-test between two sample groups. (XLSX 13 kb)

Additional file 4: **Table S4.** Differentially expressed genes between pre-concurrent chemoradiation therapy (CCRT) and post-CCRT (XLSX 1920 kb)

Additional file 5: **Table S5.** The difference between groups for relative fractions of major immune cell types compared by surgical pathology. The significance was measured by t-test between two sample groups. (XLSX 12 kb)

Additional file 6: **Table S6.** Differentially expressed genes between of pre-concurrent chemoradiation therapy (CCRT) and post-CCRT in no pCR and pCR patients (XLSX 3700 kb)

Additional file 7: **Figure S1.** Heatmap comparison of differentially expressed genes between pre- and post-concurrent chemoradiation in the non-pathologic complete response sample group. (PDF 499 kb)

Additional file 8: **Figure S2. (a)** comparison between samples from the patients who showed disease progression (PD) to neo-adjuvant chemotherapy (*n* = 2) and the samples from the patients (Other) who received surgery (*n* = 25). **(b)** Specific immune cells which showed significant higher fraction compared between the neoadjuvant chemotherapy PD patients and other patients (PDF 543 kb)

## Abbreviations

5-FU: 5-fluorouracil
CCF: cancer cell fraction
CCRT: concurrent chemoradiation therapy
CYT: cytolytic activity
DFS: disease free survival
ESCC: esophageal squamous cell carcinoma
gDNA: genomic DNA
GSEA: gene set enrichminet analysis
HLA: human leukocyte alleles
IC: inhibitory concentration
MHC: major histocompatibility complex
OS: overall survival
PCR: polymerase charin reaction
PD-L1: programmed cell death-ligand 1
PFS: progression-free survival
RNA-seq: RNA sequencing
SCC: squamous cell carcinoma
SNVs: single nucleotide variants
TMB: tumor mutation burden
WES: whole-exome sequencing
WTS: whole-transcriptome sequencing
