# Supplementary figures and images for: Paired whole exome and transcriptome analyses for the Immunogenomic changes during concurrent chemoradiotherapy in esophageal squamous cell carcinoma
Source: J Immunother Cancer. 2019 May 16;7:128. doi: 10.1186/s40425-019-0609-x (PMC6524245; doi:10.1186/s40425-019-0609-x)

Additional file 7: Figure S1

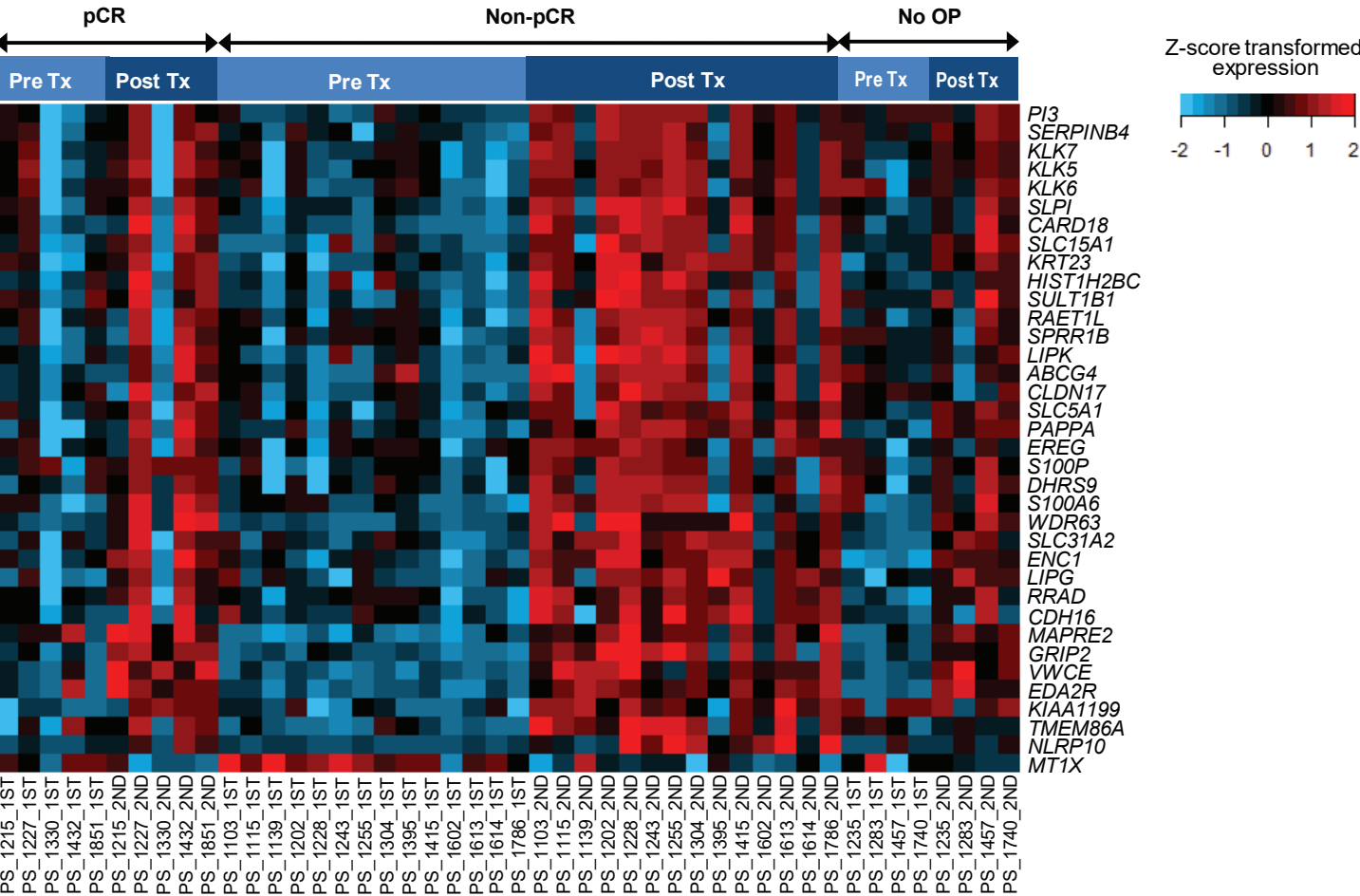

Supplement: Supplementary file 7 — Figure S1. Heatmap comparison of differentially expressed genes between pre- and post-concurrent chemoradiation in the non-pathologic complete response sample group. (PDF 499 kb) [file 40425_2019_609_MOESM7_ESM.pdf]

A

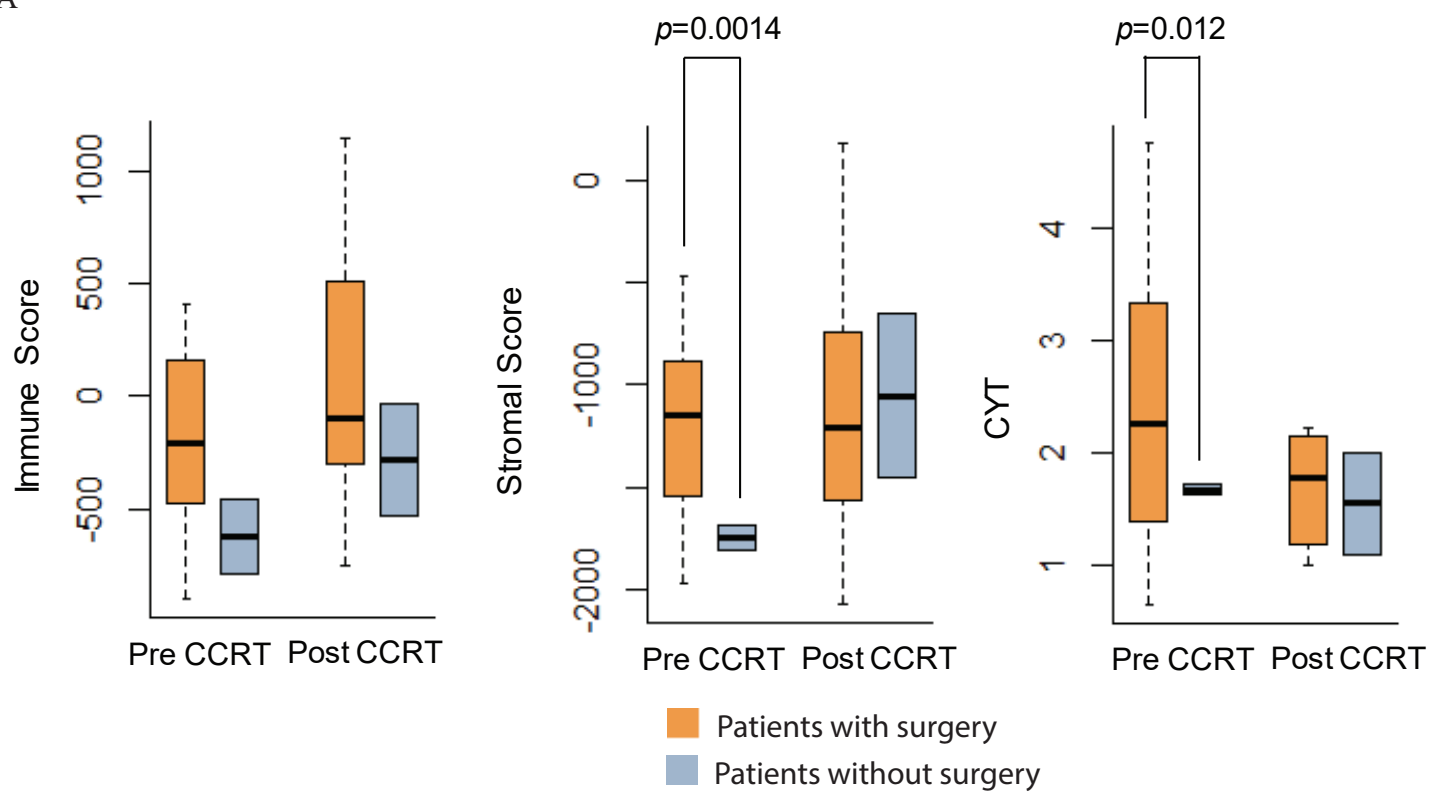

B

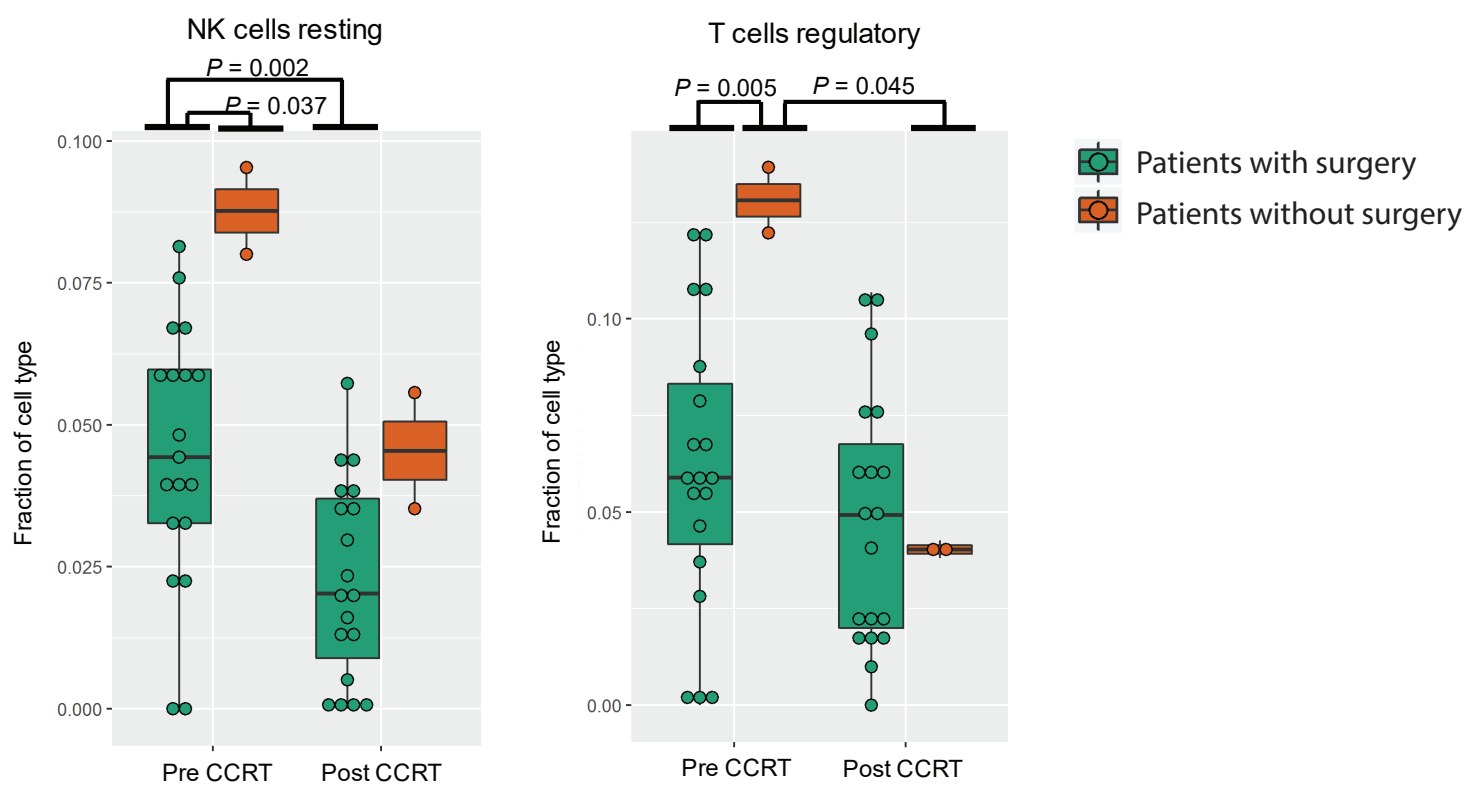

Supplement: Supplementary file 8 — Figure S2. (a) comparison between samples from the patients who showed disease progression (PD) to neo-adjuvant chemotherapy (n = 2) and the samples from the patients (Other) who received surgery (n = 25). (b) Specific immune cells which showed significant higher fraction compared between the neoadjuvant chemotherapy PD patients and other patients (PDF 543 kb) [file 40425_2019_609_MOESM8_ESM.pdf]
